# Applying augmented reality to treat Fournier’s gangrene in COVID-19 positive patients whilst safeguarding the multi-disciplinary surgical team: A case series

**DOI:** 10.1016/j.ijscr.2021.01.055

**Published:** 2021-01-19

**Authors:** Khaled Alyaqout, Shamlan AlQinai, Sulaiman Almazeedi, Jamila S. Karim, Sarah Al-Youha, Salman Al-Sabah

**Affiliations:** a Department of Surgery, Jaber Al-Ahmad Hospital, Kuwait; b Proximie, The Harley Building, 77 New Cavendish Street, London, United Kingdom

**Keywords:** Case series, Augmented reality, Fournier’s gangrene, Infection control

## Abstract

**Introduction:**

COVID-19 has presented the surgical community with a multitude of challenges. Patients requiring surgical intervention who are positive for COVID-19 are not only more likely to develop complications post-operatively, but also pose an increased infection risk to the surgical team involved in their care. The infection control concerns raised at the peak of the pandemic persist in the post-pandemic era as patients continue to test positive for COVID-19 and the risk of a ‘second wave’ looms.

**Methods:**

We present a case series (compliant with SCARE [4] and PROCESS [5] criteria) to demonstrate the effective use of an AR technology platform during the intraoperative treatment of two complex COVID positive patients diagnosed with Fournier’s gangrene.

Retrospective review of prospectively collected data of all patients that required surgery involving multiple specialties during the COVID-19 pandemic at Jaber AlAhmad hospital in Kuwait between March 2020 to October 2020.

**Presentation of case:**

We present two cases to highlight the use of an augmented reality (AR) platform during the treatment of COVID-19 positive patients with Fournier’s gangrene in order to safeguard surgical teams whilst simultaneously enabling these complex cases to benefit from multi-specialty input intraoperatively.

**Outcome:**

Augmented reality is a feasible option to minimize surgeons’ exposure during surgery without compromising the patients safety.

**Discussion:**

This case series demonstrates how AR solutions can be employed to bolster infection control measures and may be useful in the treatment of surgical patients who test positive for COVID-19.

**Conclusion:**

AR solutions could be considered as an infection control strategy to safeguard surgical teams operating on COVID-19 positive patients.

## Highlights

• Patients infected with the COVID-19 virus are at a higher risk of developing post-operative complications.
• COVID-19 patients undergoing surgical procedures pose a transmission risk to the operating room team.
• Augmented reality technology can be used to safeguard the surgical team while ensuring real-time multi-displinary expert input during the course of the procedure.
• Augmented reality technologies may form the basis of a robust infection control strategy in the post-COVID era.

## 1 Introduction

The COVID-19 pandemic has affected the delivery of surgical care on a global scale. At the height of the pandemic, surgery was reserved for the most acute, life-threatening cases. Bhangu et al., estimated the global surgical cancellation rate during the 12-week peak of the pandemic to be 72.3% and projected that 28, 404, 603 operations would be cancelled during this time [1].

As the international surgical community seeks to recover service delivery, a number of considerations need to be addressed in order to ensure that both acute and elective surgical care is provided safely [2]. Notably, integration of enhanced infection control processes will be vital to protect both patients and surgical teams from infection.

Augmented reality (AR) platforms have been used within multiple surgical specialties during the COVID-19 pandemic to bolster infection control measures by reducing the number of individuals physically required to be present during an operation and enabling surgeons to remotely access expertise intraoperatively [3]. AR technologies may form the basis of a robust infection control strategy in the post−COVID era, particularly for the treatment of COVID-19 positive patients who require surgical input from multiple specialty groups.

### 1.1 Ethical approval and registration

• Ethical approval for this study was obtained from the Kuwait Ministry of Health Ethical Review Board (Ethical approval number 1402/2020)
• Our study has been registered with Research Registry. The unique identifying number is: researchregistry6174.

## 2 Study design

Retrospective, single-centre, consecutive case series compliant with SCARE [4] and PROCESS [5] criteria.

## 3 Setting

Community Hospital dedicated for COVID-19 positive patients in Kuwait.

## 4 Case presentation

### 4.1 Case study 1

A 68-year-old, COVID-positive male was transferred to our specialist centre with a 7-day history of progressive perianal pain and swelling.

The patient was being treated for COVID-related pneumonia at the time of transfer. Although the onset of his perianal symptoms preceded his respiratory signs, he had not previously been evaluated for this issue. His medical background included hypertension, which was effectively treated with Concor.

Examination revealed a large right gluteal swelling extending to the perineum, scrotum, and superiorly along the right inguinal crease. The area of swelling was extremely tender, erythematous, hot to touch and associated with areas of crepitus. Multiple necrotic patches were also present throughout the affected area. The patient’s vital signs and blood tests were suggestive of acute infection and a CT scan confirmed findings consistent with a diagnosis of Fournier’s gangrene.

The patient was consented for surgery and extensive debridement of the gluteal region, perineum, right groin crease and scrotal skin was performed under spinal anaesthesia by a board certified general surgeon. Given the complexity of the case, the operating general surgeon used an AR platform intraoperatively to seek remote expert input from both plastics and urology services.

The patient returned to theatre every alternate day to ensure source control and subsequently made an uneventful recovery.

### 4.2 Case study 2

A 54-year-old COVID positive gentleman presented with a 3-day history of cough, fever and progressive scrotal swelling.

The patient had an extensive medical and surgical history including type II diabetes mellitus (treated with oral medication), previous toe debridement and prior surgery for a fat-containing umbilical hernia. His lower limbs were also affected due to a diagnosis of Polio during childhood, which had resulted in him being bedridden for the past two years.

On examination, the patient was found to have a tender, foul-smelling, swollen, erythematous scrotum. Necrotic patches and frank pus discharge could be appreciated at the most dependent part. There was no obvious extension to the abdominal wall or gluteal regions. A CT-scan confirmed the suspected diagnosis of Fournier’s gangrene and also revealed involvement of the perineum and right upper thigh.

The patient was consented and moved to an intensive care setting for pre-operative optimisation and intubation. Although the increased risk of general anaesthesia in COVID-19 positive patients is well recognised, it was not possible to perform the procedure under regional anaesthesia due to the patient’s previous Polio diagnosis. Extensive debridement of the affected area was performed by a board certified general surgeon with remote input from a urologist and plastic surgeon using an AR platform. The patient was transferred to ITU post-operatively and eventually stepped-down to ward-based care for wound management.

## 5 Technology and in-theatre set-up

The Proximie augmented reality platform was employed in both cases to remotely connect the operating general surgeon with a plastic surgeon and urologist intraoperatively. The technology enabled the provision of expert advice during these challenging cases without exposing additional team members to potential infection risk.

**Fig. 1:**
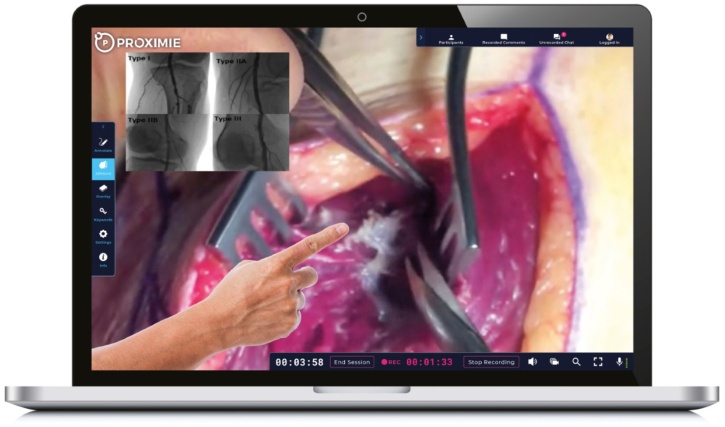
A representation of the Proximie augmented reality platform being used by a remote expert during a surgical case.

A simple laptop and webcam were utilised to capture the operative scene. The remote users accessed the live video feed through the Proximie platform using a standard laptop with a Chrome browser. The platform enabled the remote surgeons to guide the operating surgeon using voice, telestration and hand gestures (please refer to Fig. 1) [6].

## 6 Discussion

Ensuring the safe re-introduction of elective surgical services is a herculean task. Whilst most countries have instituted guidelines to certify that patients undergoing routine procedures are COVID-free pre-operatively [7], the surgical workforce that will be relied upon to clear the back log of elective procedures that have accumulated during the pandemic will also be performing acute operations on COVID positive patients. As such, it is of the utmost importance that robust infection control measures are instituted in order to safeguard surgical teams and ensure sustainable recovery of surgical services.

AR technologies have historically been used to enable surgeons in different locations to share expertise in order to provide patients with optimum care. The advancement in this technology has enhanced the Visio-verbal communication in these platforms and hence delivered a clinically acceptable level of interaction intraoperatively [8]. In both of our cases the input from different specialties was essential, moreover, because AR does not limit the number of participating doctors, the patient eventually benefited from a broader multidisciplinary input. This makes AR an attractive adjunct in theatre and a useful intraoperative tool in terms of team collaboration.

### 6.1 Strengths and limitation

The case series outlined in this paper demonstrates how AR solutions can be used to enhance infection control measures in the post−COVID era and ensure that complicated patient cases continue to benefit from multi-disciplinary input. COVID-19 lead to a reduction in the number of operations performed under general anesthesia and hence limited the application. Future research should further evaluate this technology through multicentric prospective studies with greater patients volume.

## 7 Conclusion

COVID positive surgical patients are complex and pose an increased infection risk to the surgical workforce. Whilst judicious use of personal protective equipment is imperative when treating this group of patients, the surgical community must also consider additional strategies to safeguard surgical teams. AR solutions can be effectively employed to seek expertise remotely for challenging cases and should be considered as an infection control strategy for the intraoperative treatment of COVID-19 patients where the involvement of multiple surgical teams is required. The role of AR may also help optimize patients outcome by facilitating specialties input intraoperatively, and attenuating any limitations caused by the need of transportation, such application should be studied to evaluate its feasibility and applicability.

## Declaration of Competing Interest

• The author Khaled Alyaqout declares there was no conflict of interest in this study.
• The author Shamlan AlQinai declares there was no conflict of interest in this study.
• The author Sulaiman Almazeedi declares there was no conflict of interest in this study.
• The author Salman Al-Sabah declares there was no conflict of interest in this study.
• The author Jamila S. Karim declares the following conflict: Dr. Karim is the Head of Research & Strategic Development at Proximie. This is a full-time paid position and she receives personal fees from Proximie. Proximie is an independent company that receives funding from personal investors and grants.

## Funding

A research grant was awarded by the Kuwait Foundation for the Advancement of Science to cover publication fees. The sponsor had no role in study design; in the collection, analysis and interpretation of data; in the writing of the report; and in the decision to submit the article for publication.

## Ethical approval

Ethical approval for this study was obtained from the Kuwait Ministry of Health Ethical Review Board (Ethical approval number 1402/2020).

## Consent

Written informed consent was obtained from the patients for publication of this case series and accompanying images. A copy of the written consent is available for review by the Editor-in-Chief of this journal on request.

## Author contribution

All authors contributed equally in the study design and data collection & interpretation. The authors have also contributed equally in writing and reviewing the papers.

## Registration of research studies

researchregistry6174 available at: https://www.researchregistry.com/browse-the-registry-home/registrationdetails/5f998d564a4332001577a684/.

## Guarantor

Dr. Salman Al-Sabah.

## Provenance and peer review

Not commissioned, externally peer-reviewed.

## CRediT authorship contribution statement

**Khaled Alyaqout:** Methodology, Project administration, Writing - original draft, Writing - review & editing. **Shamlan AlQinai:** Data curation, Writing - original draft. **Sulaiman Almazeedi:** Conceptualization, Resources. **Jamila S. Karim:** Writing - original draft, Writing - review & editing, Software. **Sarah Al-Youha:** Resources. **Salman Al-Sabah:** Project administration, Supervision, Validation, Funding acquisition.

## References

[1] COVIDSurg Collaborative (2020). Elective surgery cancellations due to the COVID-19 pandemic: global predictive modelling to inform surgical recovery plans. Br. J. Surg..

[2] COVIDSurg Collaborative (2020). Global guidance for surgical care during the COVID-19 pandemic. Br. J. Surg..

[3] Gregory A. (2020). Surgeon Saves Life With Help of Virtual Hand Half a World Away. https://www.thetimes.co.uk/article/surgeon-saves-life-with-help-of-virtual-hand-half-a-world-away-265w0bwq7.

[4] Agha R.A., Borrelli M.R., Farwana R., Koshy K., Fowler A., Orgill D.P., For the SCARE Group (2018). The SCARE 2018 statement: updating consensus Surgical CAse REport (SCARE) guidelines. Int. J. Surg..

[5] Agha R.A., Sohrabi C., Mathew G., Franchi T., Kerwan A., O’Neill N., for the PROCESS Group (2020). The PROCESS 2020 guideline: updating consensus Preferred Reporting Of CasE Series in Surgery (PROCESS) guidelines. Int. J. Surg..

[6] Proximie. Expanding Virtual Surgical Collaboration. Accessed October 2020. http://www.proximie.com.

[7] National Health Service (2020). Operating Framework for Urgent and Planned Services in Hospital Settings during COVID-19. https://covidlawlab.org/wp-content/uploads/2020/06/Operating-framework-for-urgent-and-planned-services-within-hospitals.pdf.

[8] Khor W.S., Baker B., Amin K., Chan A., Patel K., Wong J. (2016). Augmented and virtual reality in surgery-the digital surgical environment: applications, limitations and legal pitfalls. Ann. Transl. Med..

